# Prediction and Treatment of Difficult Cases in Colorectal Endoscopic Submucosal Dissection

**DOI:** 10.1155/2013/523084

**Published:** 2013-07-08

**Authors:** Yutaka Inada, Naohisa Yoshida, Munehiro Kugai, Kazuhiro Kamada, Kazuhiro Katada, Kazuhiko Uchiyama, Osamu Handa, Tomohisa Takagi, Hideyuki Konishi, Nobuaki Yagi, Yuji Naito, Naoki Wakabayashi, Akio Yanagisawa, Yoshito Itoh

**Affiliations:** ^1^Department of Molecular Gastroenterology and Hepatology, Kyoto Prefectural University of Medicine, Graduate School of Medical Science, 465 Kajii-cho, Kawaramachi-Hirokoji, Kamigyo-ku, Kyoto 602-8566, Japan; ^2^Department of Gastroenterology, Otsu City Hospital, Shiga, Japan; ^3^Department of Surgical Pathology, Kyoto Prefectural University of Medicine, Graduate School of Medical Science, Kyoto, Japan

## Abstract

*Purpose*. The aim of this study was to examine the characteristics of difficult cases and the learning curve in colorectal endoscopic submucosal dissection (ESD). *Methods*. We studied 518 colorectal tumors treated by ESD. Patients were divided into 2 groups such as the difficult ESD group and non-difficult ESD group in view of procedure time and procedure speed, respectively. The clinical features in each group were analyzed, and we also examined cases with severe fibrosis. Furthermore, we divided all cases into 5 periods according to experience of ESDs and investigated the rates of difficult and perforation cases. *Results*. In view of both procedure time and procedure speed, there were significant differences about mean tumor size, rates of severe fibrosis and perforation, and en bloc resection rate between the two groups. Severe fibrosis was detected in protruding tumors >40 mm in diameter. With respect to the learning curve, the rate of difficult and perforation cases decreased significantly in the late periods compared to the first period. *Conclusions*. Large tumor size, high rates of severe fibrosis and perforation, and low rate of en bloc resection are related with difficult ESD cases. The increasing of experiences can decrease the rate of difficult cases and perforation.

## 1. Introduction

In Japan and some other Western and Asian countries, ESD is reported to be an efficient treatment with a high rate of en bloc resection for large colorectal tumors, and ESD is less invasive than laparoscopic colectomy (LAC) [1]. ESD should be performed for tumors that are diagnosed as intramucosal cancer and shallowly invaded submucosal cancer [2, 3]. The number of colorectal ESD has increased gradually with the development of safer strategies and improvements of suitable ESD devices. However, the control of endoscopes and ESD knives are hindered in some colorectal ESD cases because the colon is winding and has many folds. Additionally, restlessness resulting from abdominal fullness and pain is related to prolonged procedure times [4, 5]. It is therefore important to predict difficult cases to prevent complications, including perforation [5, 6]. In this study, we investigated difficult ESD cases with long procedure times or slow procedure speed and examined learning curve of ESD.

## 2. Patients and Methods

**Figure 1:**
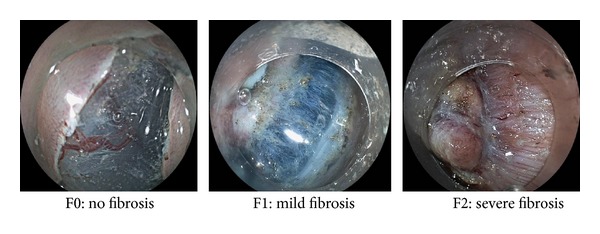
Classification about fibrosis of the submucosal layer F0: no fibrosis, manifested as a blue transparent layer, F1: mild fibrosis that appears as a white web-like structure in the blue submucosal layer, and F2: severe fibrosis that appears as a white muscle-like structure without a blue transparent layer in the submucosal layers.

A total of 518 tumors in 418 patients who underwent ESD at the Kyoto Prefectural University of Medicine or Nara City Hospital from 2006 to 2013 were analyzed. We examined clinical outcomes for all 518 tumors and divided the tumors into 2 groups such as the difficult group and non-difficult group on the basis of procedure time. The difficult group was defined as tumors that required ≥120 minutes in procedure time. On the other hand, we also divided the tumors into 2 groups such as the difficult group and non-difficult group in view of procedure speed. The procedure speed was calculated as previous reports [7]. It was calculated by dividing the procedure time into the area of the resected specimen (cm^2^/min). Briefly, the area of resected specimen was calculated as follows: 3.14 × 0.25 × long axis × minor axis. The difficult group was defined that the procedure speed was lower than the mean −1SD. In both analyses, the following factors were analyzed: tumor size, location (right-sided, left-sided colon, or rectum), morphology, the rate of cases that were performed by expert endoscopist, degree of fibrosis, complications (rate of perforation and postoperative hemorrhage), the rate of en bloc resection, and histopathological diagnosis. In view of morphology, the protruding tumor included the sessile and subpedunculated types, and the superficial tumor included the elevated, flat, and depressed types, in accordance with the Japanese Classification of Colorectal Carcinoma (JSCCR) [8]. The locations of tumors were examined among the three segments such as the right-sided colon from the cecum to the transverse colon, the left-sided colon from the descending colon to the sigmoid colon, and the rectum. Fibrosis of the submucosal layer was classified into 3 groups such as F0, F1, and F2 on the basis of the findings obtained at the time of injection of hyaluronic acid solution with indigo carmine, as previously described [9]. Briefly, the classification was as follows: F0: no fibrosis, manifested as a blue transparent layer, F1: mild fibrosis that appears as a white web-like structure in the blue submucosal layer, and F2: severe fibrosis that appears as a white muscle-like structure without a blue transparent layer in the submucosal layers (Figure 1). Histopathological diagnosis was based on JSCCR [8]. All tumors were classified as either adenoma, intramucosal cancer, or submucosally invaded cancer.

Additionally, to analyze the correlation between fibrosis and tumor size or morphology, tumors were grouped by diameter (20–29 mm, 30–39 mm, or >40 mm), and the grade of fibrosis in each group was investigated. Among superficial tumors, all laterally spreading tumors (LSTs) were further classified into 4 groups: granular homogeneous type (GH), granular nodular-mixed type (GM), nongranular flat-elevated type (NGF), and non-granular pseudo-depressed type (NGPD), according to the previously reported morphologic classification [10]. The grade of fibrosis in protruding tumor and each type of LST was investigated.

Furthermore, we examined the learning curve of the endoscopists in colorectal ESD. All cases were grouped into 5 periods on the basis of the number of procedures performed by the endoscopist: the first period (*n* < 100), the second period (*n* = 100–199), the third period (*n* = 200–299), the fourth period (*n* = 300–399), and the fifth period (*n* = 400–518). We investigated the rates of perforation cases and difficult cases in view of procedure time, mean tumor size, and procedure speed in each period.

### 2.1. ESD Procedure

The ESD procedure was performed as reported previously [5]. Our ordinal procedure was performed with short-tipped ESD knives, such as a Flush knife or a Flush knife BT (Fujifilm Medical Co., Tokyo, Japan) [7]. A Clutch cutter (Fujifilm Medical Co., Tokyo, Japan)—a grasping scissor knife—was used secondarily and added from October 2010. A lower gastrointestinal endoscope with a single channel (EC-590MP; Fujifilm Medical Co., or PCF-Q260AI; Olympus Optical Co., Ltd., Tokyo, Japan) was used. The injection solution was prepared with 0.4% hyaluronic acid solution (Mucoup; Johnson & Johnson, Tokyo, Japan or Seikagaku Corporation, Tokyo, Japan) including small quantities of indigo carmine. Mucosal injection to elevate the submucosa was performed with a 25-gauge needle (TOP Co., Tokyo, Japan). The VIO300D high-frequency generator was used in this study (Erbe Elektromedizin, Tubingen, Germany).

The six endoscopists who participated in this study were divided into expert and non-expert according to the number of colorectal ESD cases which they had performed. One endoscopist was classified as expert (having performed more than 50 colorectal ESD cases) and the other five endoscopists were classified as non-experts (having performed fewer than 50 ESD cases). Non-experts performed ESD properly with a help by the expert.

### 2.2. Statistical Analysis

All data were analyzed by using the chi-square test, Student's *t*-test, and Fisher's exact tests. *P* values of <0.05 were considered to be statistically significant.

## 3. Results

The mean tumor size, procedure time, and procedure speed for all 518 lesions were 31.0 ± 13.7 mm, 93.6 ± 55.9 minutes, and 0.10 ± 0.07 cm^2^/min, respectively (Table 1). The rate of severe fibrosis (F2) was 9.5%. En bloc resection was achieved in 91.5% of cases. Related to histopathological diagnosis, 42.1% were intramucosal cancer and 12.1% were submucosally invaded cancer.

In view of procedure time, there were significant differences about mean tumor size, rates of severe fibrosis (F2), and en bloc resection rate between the difficult group and non-difficult group (Table 2). Additionally, rates of perforation and post operative hemorrhage in the difficult group (11.0% and 6.0%) were significantly higher than those in the non-difficult group (2.2% and 1.4%).

In view of procedure speed, mean procedure speed of all tumors was 0.10 cm^2^/min. The difficult group was defined as tumors with procedure speed ≤0.03 cm^2^/min. There were significant differences about rates of severe fibrosis (F2) and en bloc resection between the difficult group and non-difficult group. The mean tumor size in the difficult group was significantly smaller than that in the non-difficult group (Table 3). Additionally, rate of perforation in the difficult group (9.6%) was significantly higher than that in the non-difficult group (3.0%).

**Figure 2:**
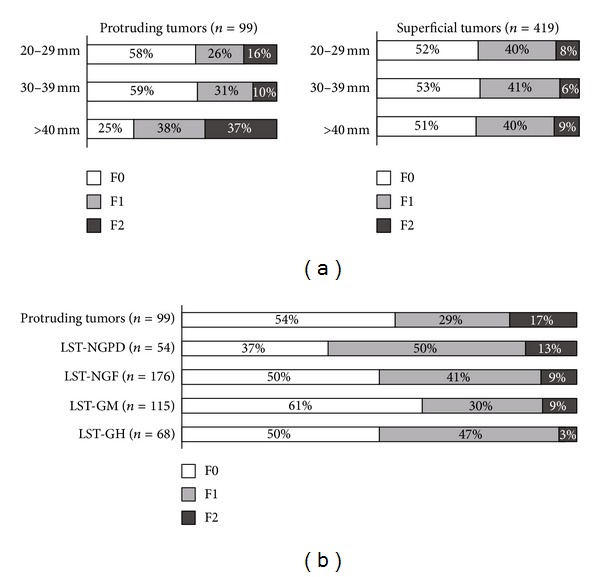
Clinical features of tumors with mild and severe fibroses. (a) There was a significantly higher incidence of severe fibrosis in protruding tumors >40 mm in diameter. However, there was no significant difference in the incidence of fibrosis in superficial tumors. (b) The incidence of severe fibrosis (F2) was higher in protruding tumors and LST-NGPD. F0: no fibrosis, F1: mild fibrosis, F2: severe: fibrosis, LST: laterally spreading tumor, NGPD: nongranular pseudodepressive type, NGF: nongranular flat type, and GM: granular nodular-mixed type, GH: granular homogenous type.

In the analysis of the relationship between fibrosis and tumor morphology, there was a significantly higher incidence of severe fibrosis in protruding tumors >40 mm in diameter (Figure 2). However, there was no significant difference in the incidence of fibrosis in superficial tumors. Furthermore, among protruding tumor and 4 types of LSTs, the incidence of severe fibrosis (F2) was higher in protruding tumor and LST-NGPD. However, there were no significant differences between the groups.

**Figure 3:**
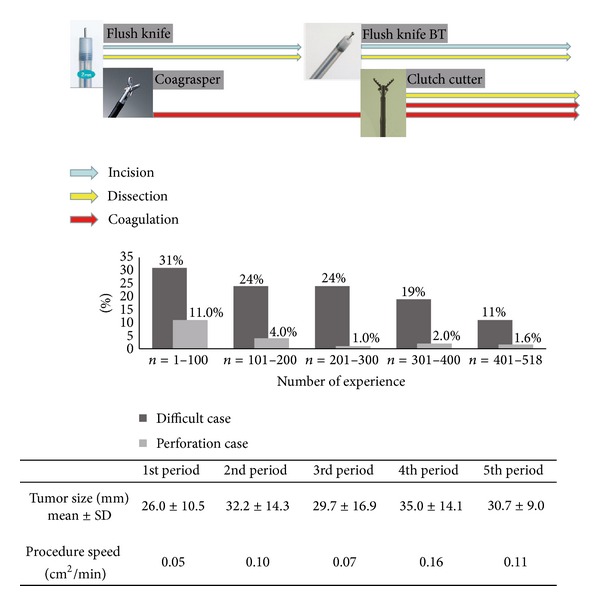
The change of ESD knife and forceps and learning curve for colorectal ESD. The rates of difficult cases in view of procedure time and perforation cases, tumor size, and procedure speed in 5 periods according to the experiences of colorectal ESD. With respect to the endoscopists' experience, the rates of difficult cases and perforation decreased with more experience.

With respect to the endoscopists' experiences, the rates of difficult and perforation cases decreased with more experience (Figure 3). And procedure speed was higher in 4th and 5th periods. The rate of difficult cases in the 4th-5th period was significantly reduced compared to the 1st period (31% versus 19%, *P* < 0.05; 31% versus 11%, *P* < 0.05). Additionally, the rates of peroration case in the 3rd-4th periods were significantly lower than those in the 1st period (11.0% versus 1.0%, *P* < 0.05; 11.0% versus 2.0%, *P* < 0.05; 11.0% versus 1.6%, *P* < 0.05).

## 4. Discussion

In this study, we demonstrated the overall therapeutic results of 518 ESD cases. The rates of en bloc resection and average procedure time were 91.5% and 93.6 minutes. ESD required for longer time than EMR, but the rate of en bloc resection was highly better than that of EMR [3]. As for complications, rates of perforation and postoperative hemorrhage were 3.8% and 2.3% similar to other reports [5–7, 11, 12].

Regarding with difficult cases in view of procedure time and procedure speed, the rate of severe fibrosis in the submucosal layer was higher in the difficult group than in the non-difficult group. In the analysis about the relationship between fibrosis and tumor morphology, a high rate of severe fibrosis was observed in protruding tumors >40 mm in diameter and in LST-NGPD. On the other hand, regarding the learning curve of ESD, the reduction in the number of perforation and difficult cases was achieved with increasing experience.

**Figure 4:**
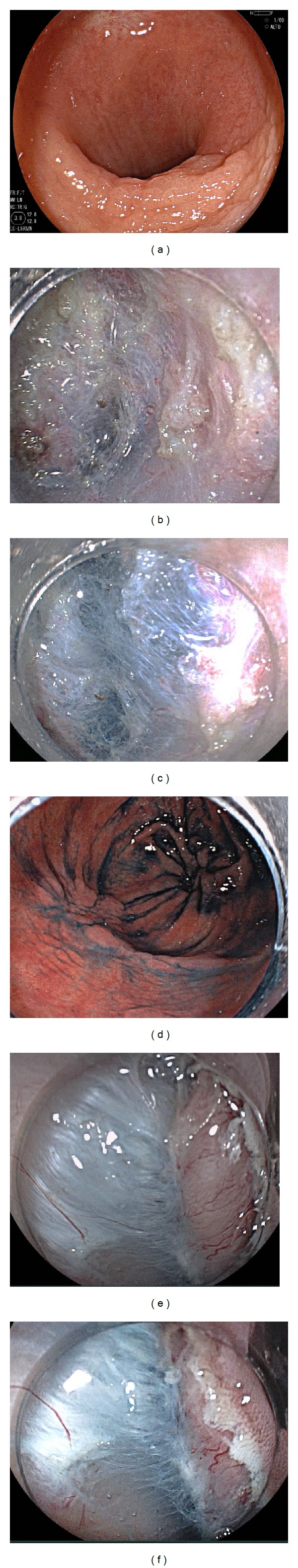
(a) Superficial tumor 60 mm in diameter with ulcerative colitis. (b) Severe fibrosis was detected in the submucosal layer. (c) Blue layer was seen in severe fibrosis after injection. (d) Recurrent lesion 30 mm in diameter after endoscopic mucosal resection. (e) Severe fibrosis was detected in the submucosal layer. (f) Blue layer was seen in sever fibrosis after injection.

Related with complications, Saito et al. [11] reported that a tumor size ≥50 mm was an independent risk factor for complications. Kim et al. [12] demonstrated that the endoscopic size of the tumor and tumor location were significantly associated with perforation. Lee et al. [13] reported that LSTs, tumor location (right-sided colon), and submucosal injection without hyaluronic acid were associated with a higher frequency of perforation. On the other hand, Matsumoto et al. [9] showed that in cases of lesions with severe (F2) fibrosis, the rate of complete en bloc resection was low and the perforation rate was high. They also reported that the incidence of F2 fibrosis in LST-GM was higher than for other morphologic types of LST. However, in our study, the incidence of F2 fibrosis was higher in LST-NGPD. Further analysis is expected to prove the relationship between morphology and fibrosis. Severe fibrosis were detected in patients with ulcerative colitis and recurrence after EMR (Figure 4). Therefore, it is necessary to consider not only the macroscopic characteristics of the lesion, but also the past history of the patient. As one of our knacks to dissect severe fibrosis, the use of hyaluronic acid with indigocarmine is useful [14]. Additionally, using slightly dense blue injection liquid by indigocarmine, we can identify the length to proper muscle layer to evaluate the dense of blue color in the submucosa. After injection of the liquid, blue layer is slightly seen in severe fibrosis. It allows to do safe dissection. We named this technique Blue-layer method. It is useful for the prevention of perforation. Thus, when the blue color is dense, we identify the length to proper muscle layer is long. Oppositely, when the blue color is light, we identify the length to proper muscle layer is short and should pay attention to dissect it (Figure 4).

Perforation is the primary complication of colorectal ESD, and it can cause fatal peritonitis [15]. Knife coagulation is the most common cause of perforation [6]. Short-tipped knives, such as the flush knife and flush knife BT, can cause perforation in some situations, for example, when the position of tumor became vertical with respect to the tip of knife. The paradoxical movement of the endoscope during ESD because of the winding nature of the colon causes coagulation in the muscularis propria. Moreover, a longer procedure time increases the amount of air in the abdomen, causing greater paradoxical movement of the endoscope. On the other hand, the Clutch cutter and the SB knife (Sumitomo Bakelite Co., Tokyo, Japan) such as scissor-type knives can grasp submucosal layer safely and make a dissection [16, 17]. Therefore, the indication of ESD according to the skill of the endoscopist, an appropriate ESD strategy, and the choice of a suitable knife are important in preventing perforations. In our institution, recently, the flush knife BT is used primarily because it effectively allows speedy and smooth dissection and local injections, and the clutch cutter is used secondarily when the risk of perforation is high because of poor elevation of the submucosa [18, 19].

The rates of difficult cases and perforation cases were high in the early periods, in particular within the first 100 procedures of colorectal ESD [20]. Saito et al. [11] and Lee et al. [13] showed that perforation risk was related to the number of ESD procedures performed, with a higher risk for endoscopists who had performed fewer than 100 procedures. Most cases of perforation are treated conservatively by endoscopic clipping, without the need for urgent surgical intervention. Therefore, proper endoscopic clipping should be performed when perforation is detected. ESD training using in vivo and ex vivo animal models is useful not only to study therapeutic strategy, but also for the treatment of perforation with methods such as clipping. Repeated animal model training procedures have also been demonstrated to decrease procedure time of ESD and endoscopic clipping [21, 22]. Many studies reported that this training should be acquired before treating difficult cases, particularly for inexperienced endoscopists [12, 21, 22].

## 5. Conclusions

Large tumor size, high rates of severe fibrosis and perforation, and low rate of en bloc resection rate are risk factors for difficult ESD cases. Increasing of experiences can decrease rate of difficult cases and perforation cases.

## Figures and Tables

**Table 1:** Clinical characteristics and outcomes of colorectal ESD.

Clinicopathological factors	*N* = 518
Age, mean ± SD	67.6 ± 10.0
Sex (*N* = 418) Male/female	241 (59.1%)/177 (40.9%)
Tumor size (mm), mean ± SD	31.0 ± 13.7
Tumor location (%) Right-sided/left-sided/rectum	48.8/19.7/31.5
Morphology (%) Superficial/protruding	80.9/19.1
Degree of fibrosis (%) (*n*)	53.1/37.4/9.5
F0/F1/F2	(275/194/49)
Experience of endoscopist (%) (*n*)	34.6/65.4
Expert/nonexpert	(179/339)
Procedure time (minutes), mean ± SD (range)	93.6 ± 55.9 (15–420)
Procedure speed (cm^2^/min), mean ± SD	0.10 ± 0.07
En bloc resection (%)	91.5
Histology Ad/M/SM, (%) (*n*)	45.8/42.1/12.1 (235/216/62)
Perforation (%) (*n*)	3.8 (20)
Postoperative hemorrhage (%) (*n*)	2.3 (12)

Right-sided: cecum to transverse colon, left-sided: descending to sigmoid colon, F0: no fibrosis, F1: mild fibrosis, F2: severe fibrosis, Ad: adenoma, M: intramucosal cancer, and SM: submucosal invaded cancer.

**Table 2:** Clinical outcomes of difficult and non-difficult groups in view of procedure time.

	Difficult group (≥120 min)	Non-difficult group (<120 min)	*P* value
Case numbers (%)	100 (19.3%)	418 (80.7%)	
Tumor size (mm), mean ± SD	41.4 ± 21.1	28.5 ± 9.8	<0.001
Tumor location (%) Right-sided/left-sided/rectum	43.0/18.0/39.0	50.2/20.2/29.6	NS
Morphology (%) Superficial/protruding	83.0/17.0	80.4/19.6	NS
Degree of fibrosis (%) F0/F1/F2	28.0/48.0/24.0 (28/48/24)	59.1/34.9/6.0 (247/146/25)	<0.001
The ratio of Expert/non-expert (%) (*n*)	37.0/63.0 (37/63)	33.7/66.3 (141/277)	NS
Procedure time (minutes), mean ± SD	184.7 ± 57.8	71.8 ± 24.8	<0.001
En bloc resection (%)	77.0	95.0	<0.001
Perforation (%) (*n*)	11.0 (11)	2.2 (9)	<0.001
Postoperative hemorrhage (%) (*n*)	6.0 (6)	1.4 (6)	<0.01
Histology (%) Ad/M/SM	33.0/51.0/16.0	47.8/40.8/11.4	NS

Right-sided: from cecum to transverse colon, left-sided: from descending to sigmoid colon, F0: no fibrosis, F1: mild fibrosis, F2: severe fibrosis, Ad: adenoma, M: intramucosal cancer, SM: submucosal invaded cancer, and NS: not significant.

**Table 3:** Clinical outcomes of difficult and non-difficult groups in view of procedure speed.

	Difficult group (≦0.03 cm^2^/min)	Non-difficult group (>0.03 cm^2^/min)	*P* value
Case numbers (%)	52 (10.0)	466 (90.0)	
Tumor size (mm), mean ± SD	17.2 ± 5.0	32.5 ± 13.5	<0.001
Tumor location (%) Right-sided/left-sided/rectum	36.5/25.0/38.5	50.4/18.9/30.7	NS
Morphology (%) Superficial/protruding	82.7/17.3	80.3/19.3	NS
Degree of fibrosis (%) (*n*) F0/F1/F2	34.6/42.3/23.1 (18/22/12)	55.2/36.9/7.9 (257/172/37)	<0.001
Experience of endoscopist (%) (*n*) Expert/nonexpert	26.9/73.1 (14/38)	35.4/65.6 (165/301)	NS
Procedure time (minutes), mean ± SD	136.7 ± 66.0	88.7 ± 52.6	<0.001
En bloc resection (%)	78.8	92.9	<0.001
Perforation (%) (*n*)	9.6 (3)	3.0 (14)	<0.01
Postoperative hemorrhage (%) (*n*)	5.8 (3)	1.9 (9)	NS
Histology (%) (*n*) Ad/M/SM	44.2/34.6/21.2 (23/18/11)	45.1/42.7/11.6 (210/199/54)	NS (0.07)

Right-sided: from cecum to transverse colon, left-sided: from descending to sigmoid colon, F0: no fibrosis, F1: mild fibrorsis, F2: severe fibrosis, Ad: adenoma, M: intramucosal cancer, SM: submucosal invaded cancer, and NS: not significant.
